# Supplementary material for: Distinct sub-second dopamine signaling in dorsolateral striatum measured by a genetically-encoded fluorescent sensor
Source: Nat Commun. 2023 Sep 22;14:5915. doi: 10.1038/s41467-023-41581-3 (PMC10517008; doi:10.1038/s41467-023-41581-3)
Supplement: Supplementary file 2 — Reporting Summary [file 41467_2023_41581_MOESM2_ESM.pdf]

nature portfolio

Corresponding author(s): David M Lovinger

Last updated by author(s): Jul 31, 2023

Reporting Summary

Nature Portfolio wishes to improve the reproducibility of the work that we publish. This form provides structure for consistency and transparency in reporting. For further information on Nature Portfolio policies, see our Editorial Policies and the Editorial Policy Checklist.

Statistics

For all statistical analyses, confirm that the following items are present in the figure legend, table legend, main text, or Methods section.

n/a	Confirmed	
☐	☒	The exact sample size (*n*) for each experimental group/condition, given as a discrete number and unit of measurement
☐	☒	A statement on whether measurements were taken from distinct samples or whether the same sample was measured repeatedly
☐	☒	The statistical test(s) used AND whether they are one- or two-sided<br>*Only common tests should be described solely by name; describe more complex techniques in the Methods section.*
☒	☐	A description of all covariates tested
☒	☐	A description of any assumptions or corrections, such as tests of normality and adjustment for multiple comparisons
☐	☒	A full description of the statistical parameters including central tendency (e.g. means) or other basic estimates (e.g. regression coefficient) AND variation (e.g. standard deviation) or associated estimates of uncertainty (e.g. confidence intervals)
☐	☒	For null hypothesis testing, the test statistic (e.g. *F*, *t*, *r*) with confidence intervals, effect sizes, degrees of freedom and *P* value noted<br>*Give P values as exact values whenever suitable.*
☒	☐	For Bayesian analysis, information on the choice of priors and Markov chain Monte Carlo settings
☒	☐	For hierarchical and complex designs, identification of the appropriate level for tests and full reporting of outcomes
☒	☐	Estimates of effect sizes (e.g. Cohen's *d*, Pearson's *r*), indicating how they were calculated

Our web collection on statistics for biologists contains articles on many of the points above.

Software and code

Policy information about availability of computer code

Data collection	Becker & Hickl SPCM64 v9.8 was used to collect in vivo photometry data; DEMON Voltammetry and Analysis Software v2011 was used to collect voltammetry data; Axon Instruments Clampex v9 was used to collect in vitro photometry data; Zeiss Zen Black v3 was used to acquire immunofluorescence images; Med Associates MEDPC 4 was used to acquire Pavlovian conditioning data
Data analysis	Axon Instruments Clampfit v9 & v10 was used to analyze in vitro photometry data; DEMON Voltammetry and Analysis Software v2011 was used to analyze voltammetry data; Microsoft Excel Office 365 version was used to organize/compile data from in vitro photometry experiments; GraphPad Prism v7 & v9 were used to graph and analyze all in vitro photometry and voltammetry data; custom codes for in-vivo photometry data analysis were developed on MATLAB 2018b and tested on MacOS and Windows; Raw photometry data from Becker&Hickl system was converted by custom script on Python v3.8.

For manuscripts utilizing custom algorithms or software that are central to the research but not yet described in published literature, software must be made available to editors and reviewers. We strongly encourage code deposition in a community repository (e.g. GitHub). See the Nature Portfolio guidelines for submitting code & software for further information.

Data

Policy information about availability of data

All manuscripts must include a data availability statement. This statement should provide the following information, where applicable:

- Accession codes, unique identifiers, or web links for publicly available datasets
- A description of any restrictions on data availability
- For clinical datasets or third party data, please ensure that the statement adheres to our policy

The data presented in this study are provided in the Supplementary Information/ Source Data file. Other data generated and processed by custom MATLAB script can be accessed by the codes in the Supplementary Codes.

Research involving human participants, their data, or biological material

Policy information about studies with human participants or human data. See also policy information about sex, gender (identity/presentation), and sexual orientation and race, ethnicity and racism.

Reporting on sex and gender	N/A
Reporting on race, ethnicity, or other socially relevant groupings	N/A
Population characteristics	N/A
Recruitment	N/A
Ethics oversight	N/A

Note that full information on the approval of the study protocol must also be provided in the manuscript.

Field-specific reporting

Please select the one below that is the best fit for your research. If you are not sure, read the appropriate sections before making your selection.

☒ Life sciences ☐ Behavioural & social sciences ☐ Ecological, evolutionary & environmental sciences

For a reference copy of the document with all sections, see nature.com/documents/nr-reporting-summary-flat.pdf

Life sciences study design

All studies must disclose on these points even when the disclosure is negative.

Sample size	Sample sizes for in vitro brain slice experiments and in vivo behavioral experiments were based on prior experience with the assays performed and in accordance with sample sizes published in the similar literature. (S.Lee et.al. Nature 2021). Also related experiments are shown in T.Patriarchi, Science 2018. Compared to those papers, we added 2 or 3 more mice per experiment.
Data exclusions	No data was excluded from analysis.
Replication	The behavioral experiments were run in two independent cohorts with similar, striatal region specific results. For in vitro brain slice experiments, each experiment was run multiple times with at least four mice/replicates per group. Additionally, due to the COVID-19 pandemic many of these experiments were first run in 2019/2020 and additional groups of mice run in late 2020 and 2022 with similar results.
Randomization	Animals were randomly assigned to experimental groups.
Blinding	Experimenters were initially blinded to treatment groups during data collection. Pavlovian experiment compares three different regions of the brain, so by seeing implant sites, it is naturally impossible to conduct blind data collection. However during the data analysis, the same analysis codes were consistently applied to the collected raw data.

Reporting for specific materials, systems and methods

We require information from authors about some types of materials, experimental systems and methods used in many studies. Here, indicate whether each material, system or method listed is relevant to your study. If you are not sure if a list item applies to your research, read the appropriate section before selecting a response.

Materials & experimental systems

n/a	Involved in the study
☐	☒ Antibodies
☒	☐ Eukaryotic cell lines
☒	☐ Palaeontology and archaeology
☐	☒ Animals and other organisms
☒	☐ Clinical data
☒	☐ Dual use research of concern
☒	☐ Plants

Methods

n/a	Involved in the study
☒	☐ ChIP-seq
☒	☐ Flow cytometry
☒	☐ MRI-based neuroimaging

Antibodies

Antibodies used

mouse anti tyrosine hydroxylase (millipore-Sigma Cat # MAB318, RRID: AB_2201528); AlexaFluor594-affiniPure donkey antimouse (Jackson Immunoresearch Laboratories, Cat# 715-585-151, RRID AB_2340855); Guinea Pig anti GFP (Nittobo medical, Cat# GFP-GP-Af1180, RRID AB_2571575); Goat anti guinea pig IgF Fab fragment (Nanoprobes Inc. Cat# 2055, RRID AB_2802149), biotinylated Goat anti guinea pig (Vector Labs, Cat# PK-4007, RRID AB_2336816), Goat anti mouse IgG (nanoprobes Inc., Cat# 2001, RRID AB_2877644) Alexa Fluor 488-affiniPure Donkey anti guinea pig (1:100, Jackson Immunoresearch Laboratories, Cat# 706-545-148, RRID: AB_2340472). GFP antibody (Abcam ab13970, RRID: AB_371416). Donkey anti Chicken-AlexaFluor488 (ThermoFisher, A78948, RRID: AB_2921070) DAPI Fluoromount-G (SouthernBiotech,0100-20).

Validation

Each antibody is commercially available has been validated previously in the lab, by other groups, and/or by the respective manufacturers. Research Resource Identifiers (RRIDs) are listed for each antibody.
https://www.sigmaaldrich.com/US/en/product/mm/mab318
https://nittobo-nmd.co.jp/pdf/reagents/GFP.pdf

Animals and other research organisms

Policy information about studies involving animals; ARRIVE guidelines recommended for reporting animal research, and Sex and Gender in Research

Laboratory animals

C57BL/6J mice were obtained from the Jackson Laboratory (Strain 000664) , DAT-IRES-Cre mice were obtained from the Jackson Laboratory (Strain 006660) , DAT KO mice were developed as previously described (Giros et al., 1996) and obtained from the Sara Jones laboratory at Wake Forest University. All mice were housed with 2-4 mice per cage and maintained on a 12:12 hour light cycle and ad libitum access to food and water All stereotaxic injections were conducted using sterile technique on mice at least 3 months of age.

Wild animals

No wild animals were used in this study.

Reporting on sex

Both male and female mice were used in each genetic line and all experiments

Field-collected samples

No field-collected samples were used in this study.

Ethics oversight

All procedures carried out in this study were in accordance with the NIH Guidelines for the Care and Use of Laboratory Animals and were approved by the Animal Care and Use Committee of the National Institute on Alcohol Abuse and Alcoholism.

Note that full information on the approval of the study protocol must also be provided in the manuscript.
